# Restoring Treatment Response in Colorectal Cancer Cells by Targeting MACC1-Dependent ABCB1 Expression in Combination Therapy

**DOI:** 10.3389/fonc.2020.00599

**Published:** 2020-04-23

**Authors:** Mathias Dahlmann, Rebecca Werner, Benedikt Kortüm, Dennis Kobelt, Wolfgang Walther, Ulrike Stein

**Affiliations:** ^1^Experimental and Clinical Research Center, Charité University Medicine and the Max-Delbrück-Center for Molecular Medicine in the Helmholtz Association, Berlin, Germany; ^2^German Cancer Consortium (DKTK), Heidelberg, Germany

**Keywords:** MACC1, colorectal cancer, multi-drug resistance, ABCB1, combination therapy

## Abstract

Treatment failure of solid cancers, represented by the development of drug resistance in the primary tumor or later outgrowth of drug resistant metastases, is the major cause of death for cancer patients. It represents an urgent clinical need for predictive biomarkers which indicate the success or failure of standard treatment regimens. Besides treatment prediction, interfering with cellular processes associated with drug resistance might improve treatment response by applying combination therapies. Metastasis-associated in colon cancer (MACC) 1 was identified in our group as a prognostic biomarker in human colorectal cancer, and has been established as key player, prognostic, and predictive biomarker for tumor progression and metastasis in a variety of solid cancers. Besides increased cell proliferation and motility, subsequently contributing to growth and metastatic spread of the primary tumor, MACC1 has also been shown to dysregulate apoptosis and is contributing to treatment resistance. Here we report the MACC1 dependent treatment resistance of colorectal cancer (CRC) cells to standard therapeutics like doxorubicin by upregulating ATP-binding cassette subfamily B member 1 (ABCB1) protein. Overexpression of MACC1 in CRC cells increased both its presence on the ABCB1 promoter and its transcriptional activity, resulting in elevated ABCB1 expression and thus treatment resistance to standard therapeutics. In contrast, depleting MACC1 increased intracellular drug concentrations, leading to better treatment response. We already identified the first MACC1 transcriptional inhibitors, such as lovastatin, by high-throughput screening of clinically approved small molecule drugs. These compounds inhibited cell motility *in vitro* but also restricted metastasis development in xenograft mouse models by reducing MACC1 expression. Here we report, that treating high MACC1 expressing CRC cells with a combination of statins and standard therapeutics increased the rate of cytotoxicity and resulted in higher treatment response.

## Introduction

Colorectal cancer (CRC) is the third most common cancer type worldwide and second in cancer-related deaths in Europe. About 1.8 million individuals are diagnosed with CRC annually worldwide and the disease-related mortality corresponds to about 50% (1, 2). Tumor stages at time of diagnosis negatively correlate with patient survival (3) as cell dissemination has often taken place in advanced stages of the disease, which critically limits the curation by surgery alone. In addition, resistance of the primary tumor to standard and targeted chemotherapy or the outgrowth of distant metastases are the main causes for treatment failure, defining a strong demand for alternative treatment strategies to overcome these obstacles.

Cancer cells can gain resistance to cytotoxic therapeutics by a variety of cellular mechanisms, which often lead to a resistant phenotype for multiple drugs. One general mechanism of drug resistance is the elevated expression of members of the ATP-binding cassette (ABC) transporter superfamily, comprehensively reviewed in Gottesman et al. (4). Among others, drug resistance in cancer cells is often caused by increased expression of the ABC subfamily B member 1 (ABCB1), which is also referred to as multi-drug resistance protein (MDR) 1 and permeability glycoprotein (P-gp). ABCB1 mediates increased efflux of a broad range of natural or synthetic compounds, e.g., anthracyclines or *vinca* alkaloids (5). Overcoming the multi-drug resistant phenotype by targeting ABCB1 in cancer cells at the functional or transcriptional level is a constant topic of anti-cancer research (6, 7), which will allow to re-use anthracyclines, like doxorubicin, as highly effective anti-cancer drugs in CRC (8, 9).

An emerging factor for the regulation of therapy resistance is the gene metastasis-associated in colon cancer (MACC) 1. MACC1 has been identified as a prognostic and predictive biomarker for many solid cancer types besides CRC (10, 11). Its expression in the primary tumors drives metastasis formation, allowing the stratification of high-risk patients even at early stages (10). Moreover, besides inducing metastasis formation, MACC1 expression is also associated with increased resistance to standard and targeted therapeutics in several cancer types, including CRC (12–15). After first describing the promoter region and expression regulation of MACC1 in CRC (16) we identified mevastatin as transcriptional inhibitor of MACC1 expression in a high-throughput drug screening, and confirmed the same effect for the FDA-approved lovastatin *in vitro*, as well as in xenograft mouse models for metastasis formation (17).

With this study, we want to explore the possibility of restoring the susceptibility of multi-drug resistant CRC cells to common, but efficient anti-cancer drugs like doxorubicin, by combination therapy and repositioning of drugs that target the expression of MACC1.

## Materials and Methods

### Cell Lines and Growth Conditions

Human CRC cell lines SW480, SW620, and HCT15, all originally from the American Type Culture Collection, were grown in DMEM supplemented with 10% fetal bovine serum (both Thermo Fisher Scientific). The generation of stable SW480 cell lines, with or without ectopic MACC1 expression (SW480/MACC1 and SW480/ev, respectively), was previously described (10). The genetic knock-out of MACC1 in the SW620 cell line was performed with the CRISPR/Cas technology, resulting in a guide RNA-mediated shift in the reading frame of MACC1 exon 4 (gRNA: 5′-CAC ATC AAG TTC ATC ACC GAG G-3′; SW620/ko-MACC1) and the control cell line without the transfection of guide RNA (SW620/ctrl). Alterations in the MACC1-coding region of single-cell sorted clones were confirmed by Sanger sequencing. All cells were maintained at 37°C in a humidified incubator with 5% CO_2_. All cells tested negative for mycoplasma, verified regularly using the MycoAlert Mycoplasma detection kit (Lonza). Authentication of the cell lines was performed by short tandem repeat (STR) genotyping at the Leibniz-Institute DSMZ (Braunschweig, Germany). STR genotypes were consistent with published genotypes for these cell lines.

### RNA Extraction and qRT-PCR

300,000 cells were grown in 6-well plates and total RNA was isolated using the Universal RNA Purification Kit (Roboklon, Germany) according to manufacturer's instructions. 50 ng of purified RNA were reverse transcribed in a 20 μl total reaction mixture with 1.25 μM random hexamers, 1 × RT-buffer, dNTP mixture of 1 mM each, 1 U/μl RNase inhibitor and 10 U/μl MuMLV reverse transcriptase (all Biozym). Reaction occurred at 30°C for 10 min, 50°C for 40 min, 99°C for 5 min and subsequent cooling at 4°C. The generated cDNA was subjected to gene specific qPCR using the HotStart DNA Master SYBR Green I Kit (Biozym) according to the manufacturer's instructions. The following gene specific primer sets were used: MACC1 fow: 5′-TTC TTT TGA TTC CTC CGG TGA-3′; MACC1 rev: 5′-ACT CTG ATG GGC ATG TGC TG-3′; ABCB1 fow: 5′-CCC ATC ATT GCA ATA GCA GG-3′; ABCB1 rev: 5′-GTT CAA ACT TCT GCT CCT GA-3′; G6PDH fow: 5′-ATC GAC CAC TAC CTG GGC AA-3′; G6PDH rev: 5′-TTC TGC ATC ACG TCC CGG A-3′. Each PCR reaction was performed in a total volume of 10 μl in a LightCycler 480 system (Roche). After initial denaturation at 95°C the amplification occurred within 40 cycles of denaturation (5 s; 95°C) and a combined primer annealing and elongation step (45 s; 60°C). Data analysis was performed with LightCycler 480 Software release 1.5.0 SP3 (Roche Diagnostics). Mean values were calculated from duplicates. Each mean value of the expressed gene was normalized to the respective mean amount of G6PDH. The results were obtained from three independent experiments.

### Protein Extraction and Western Blotting

300,000 cells were grown in 6-well plates and washed twice with 1x PBS. Cells were lysed with RIPA buffer (50 mM Tris-HCl; pH 7.5, 150 mM NaCl, and 1% Nonidet P-40, supplemented with complete protease inhibitor tablets; Roche Diagnostics) for 30 min on ice. Protein concentration was quantified with Bicinchoninic Acid Protein Assay Reagent (Thermo Fisher Scientific), according to the manufacturer's instructions. Lysates of equal protein concentration were separated by SDS-PAGE and transferred to PVDF membranes (Biorad). Membranes were blocked for 1 h at room temperature with 5% non-fat dry milk in TBS-T buffer (10 mM Tris-HCl; pH 8, 0.1% Tween 20, and 150 mM NaCl). Membranes were then incubated overnight at 4°C with MACC1 antibody (Sigma-Aldrich, dilution 1:1,000), ABCB1 antibody (CalBiochem, dilution 1:1,000) or β-actin antibody (Sigma-Aldrich, dilution 1:10,000), followed by incubation for 1 h at room temperature with HRP-conjugated anti-rabbit IgG (Promega, dilution 1:10,000) or anti-mouse IgG (Thermo Fisher Scientific, dilution 1:10,000). Antibody-protein complexes were visualized with WesternBright ECL HRP substrate (Advansta) and subsequent exposure to CL-Xposure Films (Thermo Fisher Scientific). Immunoblotting for β-actin served as the protein loading control. The results were obtained from three independent experiments.

### Doxorubicin Accumulation Assay

300,000 cells were grown in 6-well plates, were dissociated and resuspended in 0.3 ml DMEM containing 10% FCS. 0.1 ml cell suspension was treated with 30 μM doxorubicin for 1 h at 37°C. Cells were washed once with PBS and resuspended in DMEM w/o phenol red (Invitrogen) for another 3 h. Quantification of intracellular doxorubicin was performed on a LSR II flow cytometer (BD Biosciences) and analyzed by FlowJo (BD Biosciences). The results were obtained from three independent experiments. Geometric means were normalized to time point 0 and the respective parental cell line.

### Cell Viability Assay

5,000 cells were plated into 96-well plates and were allowed to accommodate for 24 h before the treatment started for 48 h. Cell viability was determined with 2 h incubation at a final concentration of 0.5 mg/ml 3-(4,5-dimethylthiazol-2-yl)-2, 5-diphenyltetrazolium bromide (MTT; Sigma-Aldrich), solubilizing the formazan crystals formed by metabolic active cells with DMSO, and measurement of the absorption at 560 nm. The results were obtained from three independent experiments. After background subtraction (day 0) results were normalized to their absorption of solvent treated controls (=100%).

### Transfection and ABCB1 Promoter Activity Assay

The ABCB1 promoter-driven luciferase reporter construct pMDR1-1202 was a gift from Kathleen Scotto (Addgene plasmid #37627; http://n2t.net/addgene:37627; RRID: Addgene_37627) (18). Plasmid transfections were carried out in 6-well culture plates using TransIT-2020 (Mirus Bio) according to manufacturer's instructions. Briefly, 250,000 cells of the cell lines SW480 and HCT15 were plated per well (three wells each) and transfected with 2 μg of the pMDR1-1202 reporter construct. DNA to lipid ratio of 1:3 was used for all the experiments. Twenty-four hours after transfection, cells were treated for further 24 h as indicated and luciferase activity was measured according to manufacturer's protocol using the Steady-Glo Luciferase Assay System (Promega) with a luminometer (Tecan infinite 200 PRO) and normalized to protein concentration.

### Chromatin Immunoprecipitation Assay (ChIP)

SW480/ev and SW480/MACC1 cells (2 × 10^6^) were plated in 10 cm dishes and grown for 24 h. Cells were cross-linked with 1% formaldehyde, lysed and sonicated to release chromatin. ChIP assay was performed using the EZ ChIP kit according to the manufacturer's instruction (Sigma-Aldrich). The protein-DNA complexes were precipitated using polyclonal antibodies for RNA polymerase II (Santa Cruz) and MACC1 (Sigma-Aldrich), or by immunoglobulin G (Sigma-Aldrich) as negative control. The extracted DNA was subjected to PCR (28 cycles at 95°C for 30 s, 60°C for 30 s, 72°C for 1 min) with two sets of ABCB1 promoter primers: S2 (−989/−747) forward 5′-AGT GGA AAC ATC CTC AGA CTA TGC-3′, reverse 5′-CCT GTC CAC TAT TTA CTT CAA ACT GAG G-3′; and S4 (−619/−363) forward 5′-CGG GCA TTG ATC TGA CGT CTG AAG TT-3′, reverse 5′-CTC CGA CCT CTC CAA TTC TGT ATC ACC T-3′ (19). ChIP assays were performed two independent times.

### Statistical Analysis

Results are presented as mean ± standard deviation from three independent experiments. Data normalization and statistical analysis was performed with the Prism software version 6.01 (GraphPad Software). Significant differences were analyzed using unpaired Student's *t*-test or one-way ANOVA followed by Bonferroni's *post-hoc* test. *p* < 0.05 was considered to be statistically significant.

## Results

### MACC1 Induces ABCB1 Expression in CRC Cell Lines

**Figure 1:**
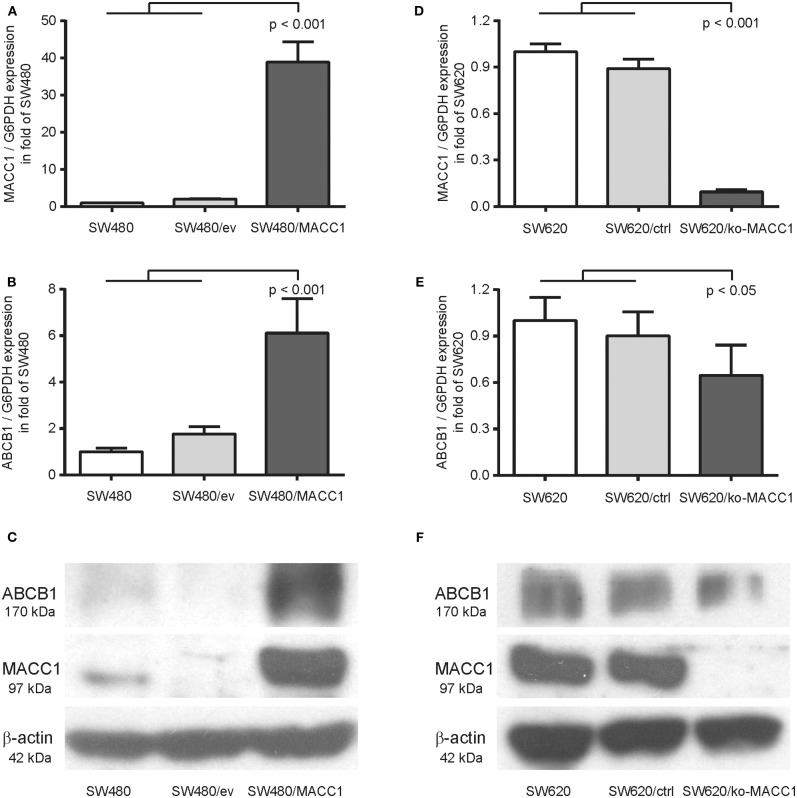
ABCB1 expression is modulated by MACC1 in CRC cell lines. **(A)** Relative expression of MACC1 in SW480 cells, with or without ectopic MACC1 expression. **(B)** Relative expression of ABCB1 in SW480 cells, with or without ectopic MACC1 expression. **(C)** Western blot of MACC1, ABCB1, and β-actin in SW480 cells, with or without ectopic MACC1 expression. **(D)** Relative expression of MACC1 in SW620 cells, with or without MACC1 knock-out. **(E)** Relative expression of ABCB1 in SW620 cells, with or without MACC1 knock-out. **(F)** Western blot of MACC1, ABCB1, and β-actin in SW620 cells, with or without MACC1 knock-out. Gene expression values were determined by gene specific qRT-PCR, normalized by G6PDH expression, and protein expression levels by Western blotting.

Previous analysis of the MACC1-dependent transcriptomic changes in the CRC cell line SW480, comparing MACC1-overexpressing SW480/MACC1 cells with their transfection control SW480/vector, resulted in significant differential expression of 1382 genes (20). Among the 656 upregulated genes we found ABCB1 almost 15-fold overexpressed in SW480/MACC1 cells, indicating a MACC1-dependent ABCB1 expression regulation. We confirmed this result in the same SW480-derived cell panel with ectopic MACC1 expression. The almost 40-fold overexpression of MACC1 in SW480/MACC1 cells compared to their control cells SW480 and SW480/ev (Figure 1A), resulted in a more than 6-fold increase in ABCB1 expression on mRNA levels (*p* < 0.001; Figure 1B). This result was confirmed on protein levels of MACC1 and ABCB1 (Figure 1C).

We tested the hypothesis of MACC1 as a regulating factor for ABCB1 expression by knocking-out MACC1 in the SW620 cell line, with high endogenous MACC1 expression. The resulting cell line SW620/ko-MACC1 harbors a homozygous frame shift in the coding sequence of MACC1, precisely at the binding site for the gene-specific primer set. The loss of MACC1 in SW620/ko-MACC1 (Figures 1D,F) reduced the ABCB1 expression on mRNA-level to about 70%, compared to the control cells SW620 and SW620/ctrl (*p* < 0.05; Figure 1E). Although ABCB1 is very weakly expressed in parental SW620 cells (21, 22) we were able to detect a decrease in the protein level of ABCB1 also in the SW620/ko-MACC1 cells, compared to SW620 and SW620/ctrl cells (Figure 1F).

### MACC1 Expression Decreases Intracellular Drug Levels and Increases Drug Resistance

**Figure 2:**
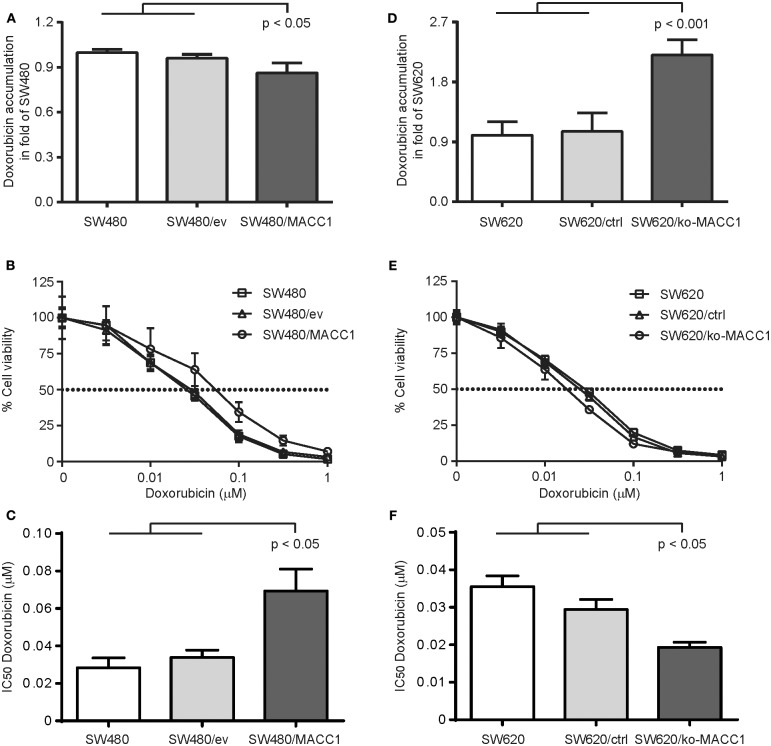
MACC1 expression decreases doxorubicin accumulation and increases treatment resistance of CRC cells. **(A)** Accumulation of doxorubicin in SW480 cells, with or without ectopic MACC1 expression, 3 h after treatment. **(B)** Cell viability of SW480 cells, with or without ectopic MACC1 expression, after 48 h of doxorubicin treatment. **(C)** IC_50_ values of doxorubicin treatment of SW480 cells, with or without ectopic MACC1 expression. **(D)** Accumulation of doxorubicin in SW620 cells, with or without MACC1 knock-out, 3 h after treatment. **(E)** Cell viability of SW620 cells, with or without MACC1 knock-out, 48 h after doxorubicin treatment. **(F)** IC_50_ values of doxorubicin treatment of SW480 cells, with or without ectopic MACC1 expression. Drug accumulation was determined by flow cytometry. Cell viability was determined by MTT assays.

As the expression of ABC transporters in general increases the efflux of therapeutic drugs, and thus limits treatment efficacy, we tested the ability of the two CRC cell panels with differential MACC1 expression to regulate intracellular accumulation of doxorubicin. Cells were transiently treated with doxorubicin and intracellular drug concentration was determined by flow cytometry (23, 24). Intracellular doxorubicin-derived fluorescence was decreased by 20% in SW480/MACC1 cells 3 h after drug exposure, compared to SW480 control cells (*p* < 0.05, Figure 2A). Nevertheless, this contributed to an increased drug resistance in SW480/MACC1 cells. By profiling cell viability after 48 h of doxorubicin treatment with increasing concentrations (Figure 2B), we observed a nearly doubled IC_50_ value for SW480/MACC1 cells of 69.4 nM ± 11.6 (*p* < 0.05; Figure 2C), compared to SW480 and SW480/ev cells (28.4 nM ± 5.3 and 33.9 nM ± 3.9, respectively).

In contrast, SW620/ko-MACC1 cells retained twice as much intracellular doxorubicin, compared to SW620 and SW620/ctrl cells (*p* < 0.001, Figure 2D), 3 h after doxorubicin treatment. In line with this finding, SW620/ko-MACC1 cells became more susceptible to the cytotoxic effect of doxorubicin (Figure 2E), compared to SW620 and SW620/ctrl cells. We determined the IC_50_ value for SW620 and SW620/ctrl cells with 35.5 nM ± 2.9 and 29.4 nM ± 2.7, respectively, and observed a significantly decreased IC_50_ value for SW620/ko-MACC1 cells of 19.3 nM ± 1.4 (*p* < 0.05; Figure 2F), representing 55 and 65% of the respective controls.

### Lovastatin Decreases the Expression of MACC1 and ABCB1 in CRC Cells and Reduces Chemoresistance to Doxorubicin

**Figure 3:**
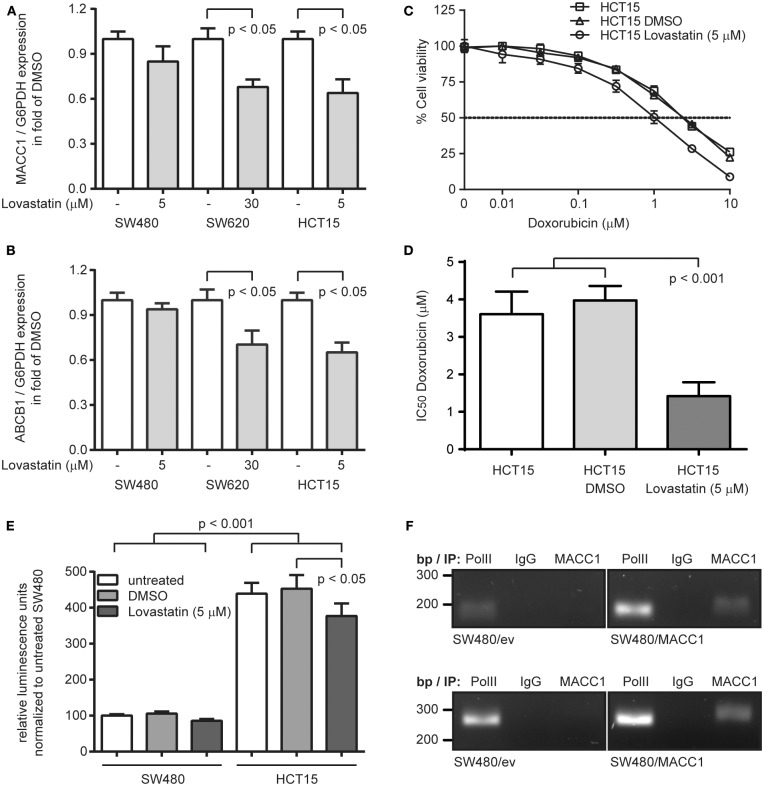
Lovastatin indirectly increases treatment response to doxorubicin, by reducing the MACC1-dependent ABCB1 expression in MACC1 expressing CRC cells. **(A)** Relative expression of MACC1 in SW480, SW620, and HCT15 cells, with or without lovastatin treatment of the indicated concentration. Solvent-treated cells were used as controls. **(B)** Relative expression of ABCB1 in SW480, SW620, and HCT15 cells, with or without lovastatin treatment of the indicated concentration. Solvent-treated cells were used as controls. **(C)** Cell viability of HCT15 cells, with or without combinatorial treatment of lovastatin and doxorubicin. **(D)** IC_50_ values of doxorubicin treated HCT15 cells, with or without combinatorial treatment of lovastatin. Gene expression was determined by qRT-PCR, and cell viability by MTT assays. **(E)** Relative luminescence signals of SW480 and HCT15 cells after transfection with an ABCB1 promoter-driven luciferase-reporter construct, with or without lovastatin treatment. **(F)** Chromatin immunoprecipitation of MACC1 in SW480/MACC1 and SW480 control cells and probing for the ABCB1 promoter regions −989 bp to −747 bp (upper panel) and −619 bp to −363 bp (lower panel). Pulling down RNA polymerase II (PolII) and unspecific human immunoglobulin G (IgG) served as positive and negative controls, respectively.

With the recent identification of statins as transcriptional inhibitors of MACC1 expression, we aimed at targeting ABCB1 expression by statin-mediated inhibition of MACC1 transcription. We treated SW480 and SW620 cells with lovastatin (5 and 30 μM, respectively) for 48 h, but also included the CRC cell line HCT15 (5 μM), which has been reported to endogenously express elevated ABCB1 levels and thus shows increased multi-drug resistance, but also expresses considerable amounts of MACC1 (21, 25, 26). As expected by the very low levels of MACC1 in SW480 cells we did not observe a significant change in MACC1 expression upon lovastatin treatment (Figure 3A). But in line with our previous results (17) we found a reduction of MACC1 expression in lovastatin-treated SW620 cells to 70% of solvent treatment (*p* < 0.05; Figure 3A). Treatment of HCT15 cells resulted in a similar decrease of MACC1 expression, compared to solvent treated cells (*p* < 0.05; Figure 3A). The same pattern in differential expression of MACC1 upon lovastatin treatment was seen for ABCB1. SW480 cells showed no significant changes in ABCB1 expression under lovastatin treatment, when compared to solvent controls (Figure 3B). Interestingly, treatment of SW620 cells with lovastatin resulted in a significantly lower ABCB1 levels, compared to solvent treated cells, which was comparable to the decrease of ABCB1 expression in lovastatin-treated HCT15 cells (Figure 3B).

Next, HCT15 cells were subjected to combination treatment with 5 μM lovastatin and increasing concentrations of doxorubicin, determining cell viability after 48 h (Figure 3C). In line with the reported multi-drug resistance of HCT15 cells, we determined an IC_50_ value of 3.61 μM ± 0.60 for the treatment with doxorubicin alone. Additional solvent-treatment resulted in a similar IC_50_ value of 3.97 μM ± 0.39. However, combination treatment of lovastatin with doxorubicin reduced the IC_50_ value to 1.42 μM ± 0.37 (*p* < 0.001; Figure 3D), clearly sensitizing the lovastatin-treated cells to the cytotoxic effect of doxorubicin.

### MACC1 Drives ABCB1 Expression at the Promoter Level

Next, we investigated the potential mechanism of the MACC1-dependent regulation of ABCB1 expression. First, we confirmed the induction of ABCB1 transcription with a luciferase-based reporter assay for the ABCB1 promoter (18). Transfection of SW480 cells with the reporter construct resulted in rather low luminescence, which did not significantly change with treatment of the cells with 5 μM lovastatin (Figure 3E). In contrast, the luciferase activity was more than 4-fold increased when assayed in HCT15 cells (*p* < 0.001; Figure 3E). In addition, the luminescence signal in HCT15 cells was significantly decreased upon lovastatin treatment (*p* < 0.05; Figure 3E).

With the focus on the ABCB1 promoter, we performed chromatin immunoprecipitation experiments in SW480 control cells, as well as SW480/MACC1 cells, and probing for co-precipitated regions of the ABCB1 promoter (−989 bp to −747 bp and −619 bp to −363 bp, respectively) (19). In general, the occupation of the ABCB1 promoter by RNA polymerase II (PolII) was lower in SW480 control cells, compared to SW480/MACC1 cells (Figure 3F) which reflects the initial ABCB1-mRNA expression data (Figures 1A,B). Pulling down MACC1 in SW480/MACC1 cells resulted in the co-precipitation of the ABCB1 promoter, which was neither observed in SW480 control cells nor by pulling down with human immunoglobulin G (IgG) (Figure 3F).

## Discussion

Multi-drug resistance is a major obstacle in the success of anti-cancer chemotherapy. In the case of CRC, upregulation of ABC transporters with broad substrate specificity during therapy critically limits the use of common and effective cytotoxic drugs like anthracyclines (27). To meet the clinical need to overcome multi-drug resistance, current research concentrates on small molecules, either natural or synthetic compounds, which can functionally inhibit ABC transporters or reduce their induction under therapy (28–33). In addition, the focus on approved drugs in overcoming multi-drug resistance allows the fast translation of novel treatment strategies into the clinics.

In this study, we report the re-sensitization of multi-drug resistant CRC cells to doxorubicin treatment, by repurposing lovastatin to interfere with the MACC1-dependent expression regulation of ABCB1. Initial hints of transcriptional regulation of ABCB1 by MACC1 were obtained from microarray analyses comparing MACC1-overexpressing SW480 cells with their transfection controls, which was independently reported for generated 5-FU-resistant CRC cell lines (15, 20). We confirmed the role of MACC1 in modulating ABCB1 expression in two CRC cell panels with differential MACC1 expression levels, and found corresponding de- and re-sensitization to doxorubicin treatment upon ectopic expression or knocking-out of MACC1, respectively. By luciferase-based reporter assays and ChIP experiments, we found MACC1 acting on the promoter of ABCB1, thereby increasing its expression. Potential regulatory mechanisms of ABCB1 expression contain constitutive promoter up- or downregulation, as well as stress/drug induced expression regulation or the improved survival of chromosomal rearrangements or mutations of the *ABCB1* gene (34). A more distal region of the ABCB1 promoter shown to increase ABCB1 expression, contains a binding site for AP-1 and a CCAAT-box, and its transcriptional activity is regulated by histone acetylation/deacetylation (35). This region also contains several binding sites for TCF-4/β-catenin transcriptional complexes (36). The presence of MACC1 on promoters regulating cancer progression and metastasis has previously been reported (10, 37, 38). But also the impact of MACC1 expression on β-catenin-dependent target gene expression was shown for CRC cells (39). Both aspects lead to the model of activating MACC1-containing protein (sub-) complexes of TCF-4/β-catenin-driven target gene transcription. With *ABCB1* as such a target gene, the overexpression of MACC1 in cells with activated Wnt/β-catenin signaling leads to increased resistance to anti-cancer therapies, including anthracyclines like doxorubicin.

The use of anthracyclines in the treatment of metastasized and refractory CRC regained attention and is currently evaluated in a phase II clinical trial (40). But also combination therapy including doxorubicin has been successfully applied to treat unresectable colorectal liver metastases (41, 42). The formation of liver metastases is observed in ~50% of CRC patients (43). The expression of MACC1 in CRC correlates with the occurrence of liver metastases (44) and its role in liver metastasis has been proven in various animal models (10, 20, 45, 46). Targeting MACC1 expression in CRC cells with the transcriptional inhibitors rottlerin and lovastatin significantly reduced metastasis formation to the liver in animal models (17), and gave rise to the idea to also combat MACC1-modulated multi-drug resistance in CRC. Lovastatin has already been reported to increase the effects of anti-cancer treatment in CRC cells (47, 48) and improves the efficacy of doxorubicin in animal models of several cancers, including colon cancer (49, 50).

With the promising results of this study, in overcoming multi-drug resistance in CRC cells by combination therapy of lovastatin and doxorubicin, it will be worth to explore further combination strategies for novel anti-tumor but also anti-metastatic therapies of CRC in pre-clinical models. Since both drug classes have proven their efficacy also in cancer therapy (51, 52), a rapid translation of combination strategies to combat treatment resistance into the clinics is feasible.

## Data Availability Statement

The datasets generated for this study can be found in the NCBI Gene Expression Omnibus (GSE70458).

## Author Contributions

US, WW, and MD: conception and design. MD and RW: acquisition and analysis of data. BK and DK: generation of cell lines. MD: writing of the manuscript. US, WW, RW, DK, and BK: critical review of the manuscript. All authors read and approved the final manuscript.

## Conflict of Interest

The authors declare that the research was conducted in the absence of any commercial or financial relationships that could be construed as a potential conflict of interest.
